# The Association between measures of Sleepiness and Subjective Cognitive Decline symptoms in a diverse population of cognitively normal older Adults

**DOI:** 10.1002/alz.093993

**Published:** 2025-01-09

**Authors:** Anthony Q Briggs, Rebecca A Betensky, Mark A Bernard, Arjun V. Masurkar

**Affiliations:** ^1^ NYU Alzheimer’s Disease Research Center, New York, NY USA; ^2^ NYU College of Global Public Health, New York, NY USA; ^3^ NYU Grossman School of Medicine, New York, NY USA; ^4^ Alzheimer’s Disease Research Center, New York University Langone Health, New York, NY USA

## Abstract

**Background:**

Subjective cognitive decline (SCD) is a preclinical stage of Alzheimer’s disease (AD), with correlations to cerebral amyloid and tau and accelerated cognitive decline. Studies have also revealed an association between sleep fragmentation and such AD biomarkers and cognitive decline, suggesting that cognitive function should be monitored in individuals experiencing excessive sleepiness. It is unclear if and how sleep dysfunction relates to SCD apart from AD biomarkers, as well as symptoms related to SCD and sleep dysfunction, such as depression. Furthermore, studies of SCD and sleep dysfunction have primarily focused on non‐Hispanic Whites. Hence, a better understanding of how sleep impacts preclinical AD risk in SCD is needed in more diverse cohorts. We investigated the cross‐sectional relationship between excessive sleepiness in cognitively normal older adults and SCD symptoms linked to cognitive domains in a cohort consisting of NHWs and Black/African Americans (B/AA).

**Method:**

This retrospective cross‐sectional study included cognitively normal older participants (n = 147, age> 60 years, 61 B/AA, 86 NHW) evaluated at the NYU Alzheimer’s Disease Research Center. Participants underwent Uniform Data Set 3.0 psychometric testing to confirm normal cognition, Epworth Sleepiness Scale (ESS), Geriatric Depression Scale (GDS), and the Cognitive Change Index (CCI) as a measure of SCD. The CCI was further divided into subscores of non‐amnestic (i.e. executive) and amnestic domains (i.e. memory). Participants underwent amyloid (florbetaben) and tau (PI‐2620 or MK‐6240) PET MRI, with composite SUVR calculated by averaging key cortical regions. Multivariable linear regression models tested the association of ESS and covariates (GDS, amyloid SUVR, tau SUVR, race) to the three CCI outcome measures (total, non‐amnestic, amnestic).

**Result:**

ESS (excessive sleepiness) was significantly positively associated with total CCI (p = 0.01042) as well as non‐amnestic (0.00771) and amnestic CCI subgroups (0.02583), after full covariate adjustment, as was GDS. Race did not show an independent statistically significant association.

**Conclusion:**

These results suggest that excessive sleepiness, independent of depression and amyloid and tau burdens, may be a significant contributor to SCD severity. Furthermore, these contributions impact both amnestic and non‐amnestic domains. Future studies are needed to investigate the longitudinal impact of excessive sleepiness on SCD outcomes.

